# A bacteria-based carbon sequestration and waste recycling system

**DOI:** 10.1038/s41598-022-14239-1

**Published:** 2022-06-28

**Authors:** Yeon Hwa La, Ki-Sung Lee, Tae-Wan Kim, Jae Yang Song

**Affiliations:** Environmental R&D Center, Institute of Environmental Science & Technology, SK Innovation, 325 Expo-ro, Yuseong-gu, Daejeon, 305-712 South Korea

**Keywords:** Biotechnology, Microbiology

## Abstract

Achieving carbon neutrality requires a variety of technological approaches. In the present study, we confirmed the applicability of a carbon cycle system in several industrial fields using sulphur-oxidising bacteria. This system produces a nitrogen fertiliser, which decreases carbon emissions by recycling H_2_S and NH_3_ pollutants discharged into the atmosphere or wastewater. It should be considered in industrial fields as a carbon reduction strategy.

## Introduction

The reduction of greenhouse gases is undoubtedly a task that should be undertaken by everyone in this era for the continuous advancement of humanity. Numerous studies have investigated ways to achieve a virtuous carbon cycle by recycling CO_2_, the greenhouse gas with the highest discharge, to replace conventional petroleum-based products. Such CO_2_ conversion technologies are broadly divided into chemical and biological conversion, including thermal catalytic, electrochemical, and photochemical conversion depending on the method of energy production, with the latter mainly involving the use of light, hydrogen, and electricity as the energy source^1^. These methods mostly rely on technological development under the condition of renewable energy supply to harness energy from sunlight, wind, and geothermal heat. However, in regions with an insufficient level of renewable energy, there is a limit to the use of such technologies to decrease CO_2_ emissions.

To overcome this problem, a novel system for the virtuous carbon cycle was proposed, whereby the chemical energy from waste resources is used to reduce CO_2_ and produce eco-friendly ammonium sulphate. The system is based on a core technology that applies sulphur-oxidising bacteria (SOB), chemolithotrophs that fix CO_2_ via the cbb pathway using reduced sulphur as the energy source. Among the three known types (acidophiles, neutrophils, and alkaliphiles), the most widely studied genus is *Acidithiobacillus*, which is applied in the biomining field^2^.

## Results

### Optimised culture conditions at a laboratory scale and CO_2_ conversion rate of SOB in continuous stirred-tank reactor (CSTR)

**Figure 1:**
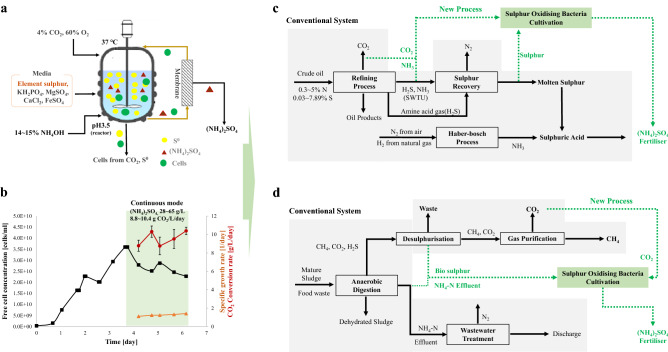
Direct CO_2_ conversion by sulphur-oxidising bacteria and its application in different industries. (**a**) Optimised culture conditions at a laboratory scale. (**b**) CO_2_ conversion rate of sulphur-oxidising bacteria (SOB) in continuous stirred-tank reactor (CSTR). (**c**) A carbon cycle system proposed in petrochemical industry and (**d**) in the anaerobic digestion industry. Black arrows indicate the conventional system and green dotted arrows indicate the new processes.

*Acidithiobacillus* can survive at a pH of 0.5 to allow the direct supply of CO_2_ from flue gas as the carbon source. Lab-scale tests showed an additional benefit of removing sulphur oxides (SOx) and a small amount of nitrogen oxides (NOx) through the bioreactor (Supplementary Fig. 1). Microbial growth is active even at a high concentration (14–15%) of ammonia solution and is used as a pH regulator, making it suitable for the production of biological ammonium sulphate (BAS). In an optimised continuous stirred tank reactor culture system (Fig. 1a,b), the use of bio sulphur as the energy source leads to a CO_2_ conversion rate of 8.8–10.4 g/L/D and ammonium sulphate production of 28–65 g/L. The CO_2_ conversion rate was slightly lower (5.6–6.3 g/L/D) when chemical sulphur was used as the energy source, possibly because chemical sulphur has large, hydrophobic particles that are relatively difficult for uptake by the bacteria. In contrast, the particles of bio sulphur are small and hydrophilic. The SOB used in this study was the AZ11 strain isolated from the soil for H_2_S elimination by Lee et al.^3^ Chromosomal DNA sequence analysis showed that AZ11 is a new species with less than 82% homology with known *Acidithiobacillus* species (Supplementary Fig. 2). The CO_2_ conversion rate using this new species was six to seven fold higher than that of the most well-known cyanobacteria, indicating a faster rate than any other reported strain in the continuous biological CO_2_ conversion system^4^.

### A carbon cycle system proposed in the petrochemical and anaerobic digestion industries

The virtuous carbon cycle using SOB is expected to prove useful in all industries that discharge CO_2_, reduced sulphur, or reduced nitrogen. For example, it may be applied in the petroleum industry and biological anaerobic digestion of waste resources (food, manure, agricultural by-products, and wastewater sludge) (Fig. 1c,d).

In the petroleum industry, 0.3–5% of the nitrogen contained in the crude oil is converted to NH_3_ during the refining process (Fig. 1c), followed by feeding it to the sulphur recovery plant by stripping at the sour water treatment unit. NH_3_ is mostly oxidised to N_2_ in the combustion chamber, but 50–70 ppm of 3% O_2_ dry NOx is also generated. It has recently been reported to be more economically feasible to apply NH_3_ as the feedstock in ammonia fertilisers by isolating it from sour water stripping gas via dual-stage stripping^5^. Thus, DuPont produces ammonium sulphate fertiliser by combining the recovered NH_3_ and sulphuric acid produced using sulphur gases^6^. If sulphuric acid can be produced by SOB while recovered NH_3_ is applied as the pH regulator in cultivation, an eco-friendly process that can directly capture CO_2_ with BAS production may be achieved.

The scope of application may be extended to the anaerobic digestion process to obtain biogas from biological resources or the anaerobic fermentation system to produce landfill gas (Fig. 1d). The C, N, and S in the feed are each converted to CH_4_ and CO_2_, NH_4_-N wastewater, and H_2_S, respectively, by facultative anaerobic and strictly anaerobic bacteria. H_2_S gas is produced in the range of 100–3000 ppm and is removed using the ferris-based dry desulphurising or sodium-based wet process, while these well-known commercial facilities pose no technical issues. However, dry desulphurisation leads to landfill costs for the waste treatment of used desulphurising agents, and wet desulphurisation leads to wastewater treatment costs. The Thiopaq desulphurisation process allows the collection of sulphur resources for reuse as pesticides, and despite the high cost, there is a considerable advantage of excavating additional values from bio sulphur products^7^. The level of total nitrogen produced in the effluent of anaerobic digestion is approximately 3000 mg/L, and due to the extremely low C/N ratio of 1.7–3.9, a problem arises in supplying additional organic carbon in the activated sludge process for denitrification. It is certainly possible to recover ammonia by stripping, or the production of ammonium carbonate by CO_2_ reaction may be considered depending on product demands and overall economic feasibility^8^. Overall, the conventional process focuses solely on carbon resource recovery to allow the treatment of nitrogen and sulphur waste in the cheapest process that satisfies the legal criteria. Suppose a process that maximises resource cycling by connecting CO_2_ and wasted chemical energy is selected; in that case, the system of the virtuous cycle may be established, whereby the nitrogen and sulphur obtained from nature can be converted into nitrogen fertiliser products of high added value to ultimately return to nature through agriculture.

## Discussion

Carbon sources on earth include C, H, O, N, and S, leading to the formation of CO_2_, NH_3_, NOx, H_2_S, or SOx in the process of energy production by humans. While CO_2_ can be directly released into the atmosphere, the substances (H_2_S/SOx and NH_3_/NOx) require environmental facilities to control their discharge according to the safety criteria for toxicity. Among them, the oxidised forms (SOx and NOx) are regarded as stable substances with low energy levels, but the reduced forms (H_2_S and NH_3_) have oxidising potential, and such chemical energy could be used as the energy for SOB-based CO_2_ conversion rather than being treated as waste requiring additional energy. Hence, the energy for H_2_S and NH_3_ treatment can be saved, and maximum resource utilisation can be achieved simultaneously.

Resource recycling that connects SOB and the C, N, and S waste products is yet in the proof-of-concept step; thus, further studies should develop ways to enhance the CO_2_ conversion rate to a far greater level. If the SOB growth rate and concentration were increased to 2.0/day and 20 g/L, respectively, through genetic engineering or optimisation of the pressurised culture, the predicted annual reduction of CO_2_ is approximately 10,000 tons per year, with the production of 64,000 tons of BAS, which is a volume similar to the commercial ethanol production reactor of Lanzatech (500 m^3^) (Supplementary Table 1). Moreover, a metabolomic study by Martinez et al. showed that *Acidithiobacillus* species might also lead to the production of such beneficial substances, including glutathione and aspartic acid^9^.

The produced ammonium sulphate would show a far shorter material mass flow than that shown in the conventional production system, involving molten sulphur produced through the Claus reaction in the refining process to be supplied to the merchant market, after which it is turned into sulphuric acid by the fertiliser raw material manufacturer and then used to produce ammonium sulphate through the reaction with ammonia produced in the Haber–Bosch process. As is widely known, the Haber–Bosch process is one of the largest global energy consumers and greenhouse gas emitters, responsible for 1.2% of global anthropogenic CO_2_ emissions; thus, alternative production methods are currently warranted^10^. Therefore, the concept of using the abundant by-product of sulphur as energy and NH_3_, which is conventionally combusted as waste in oil refining plants, is highly attractive, as CO_2_ reduction and production of nitrogen fertilisers containing ammonium sulphate can be simultaneously achieved.

If genetic engineering and optimised cultivation can be used to enhance the CO_2_ conversion rate, the reduction of greenhouse gases and issues related to fertilisers may be resolved to a certain degree in the regions where the use of renewable energy, such as sunlight and wind, is limited. The technology to enhance the CO_2_ conversion rate is thus anticipated to serve as a realistic, carbon–neutral solution in the future.

## Methods

### Flask cultivation

The strain *Acidithiobacillus* AZ11 was obtained from the Korean Collection for Type Cultures (KCTC). Bacterial cells from the agar plate were inoculated in 50 mL of basal medium containing 10 g/L sulphur (Bio-sulphur, Ecobio, Korea; chemical sulphur, Samchun Chemicals, Korea), 3 g/L KH_2_PO_4_, 1 g/L (NH_4_)_2_SO_4_, 0.5 g/L MgSO_4_, 0.25 g/L CaCl_2_, and 0.01 g/L FeSO_4_. The flask was then incubated at 30 °C for approximately 7 days in a 2000 ppm CO_2_ incubator.

### Continuous cultivation

Cultivations were performed in a stirred-tank reactor (Sartorius AG, Germany) with a 6-blade radial impeller without baffle. The culture broth in the flask was inoculated into the vessel (1.5 L of working volume) with a 3% inoculum. The culture medium was basal medium containing 30 g/L sulphur, 8 g/L KH_2_PO_4_, 0.5 g/L MgSO_4_, 0.25 g/L CaCl_2_, and 0.01 g/L FeSO_4_. Aeration was performed through a micro sparger at 0.3–0.6 vvm, and the agitation speed was set at 700–900 rpm depending on sulphur dispersion. The cultivation temperature was 37 °C, and the pH was maintained at 3.5 by adding 14–15% NH_4_OH to neutralise the sulphuric acid produced. The culture broth was filtered through a hollow fibre (PES, pore size 0.2 μm, Repligen Corp., USA) when the sulphate concentration reached more than 40 g/L. Products were harvested using a peristatic pump, and the culture volume was maintained by the addition of fresh medium. The partial pressures of oxygen and carbon dioxide were 0.4 bar.a and 0.06 bar.a, respectively.

### Calculating CO_2_ conversion rate

The collected products were centrifuged at 12,227 rcf for 20 min, and the organic carbon of the supernatants was analysed via a total organic carbon analyser (Shimadzu Corp., Japan). The precipitates were freeze dried, and the carbon contents were analysed via an elemental analyser (Thermo Fisher Scientific Inc., USA). The CO_2_ conversion rate was calculated using the following equation:

$$\mathrm{V}=C\times \mu \times \frac{44}{12}$$

where V is the CO_2_ conversion rate (g of CO_2_/L/day), C is the total carbon content (g/L), and μ is the specific growth rate [1/day].

### Analytical methods

Cell concentration was determined using an image-based, automated cell counter (Logos Biosystems Inc., Korea). The sulphate concentration was analysed via high-performance liquid chromatography using a Waters system consisting of a refractive index detector and a Bio-Rad Aminex-HPX-87H column (300 × 7.8 mm). The column temperature was set to 60 °C, and the detector flow cell was maintained at 45 °C. The mobile phase was 0.005 M sulphuric acid.

## Supplementary Information

Supplementary Figure 1.

Supplementary Figure 2.

Supplementary Table 1.

## Data Availability

Data used in this study is available from the corresponding author upon reasonable request.
