# Supplementary material for: A bacteria-based carbon sequestration and waste recycling system
Source: Sci Rep. 2022 Jun 28;12:10236. doi: 10.1038/s41598-022-14239-1 (PMC9240062; doi:10.1038/s41598-022-14239-1)
Supplement: Supplementary file 1 — Supplementary Figure 1. [file 41598_2022_14239_MOESM1_ESM.pptx]

## Slide 1
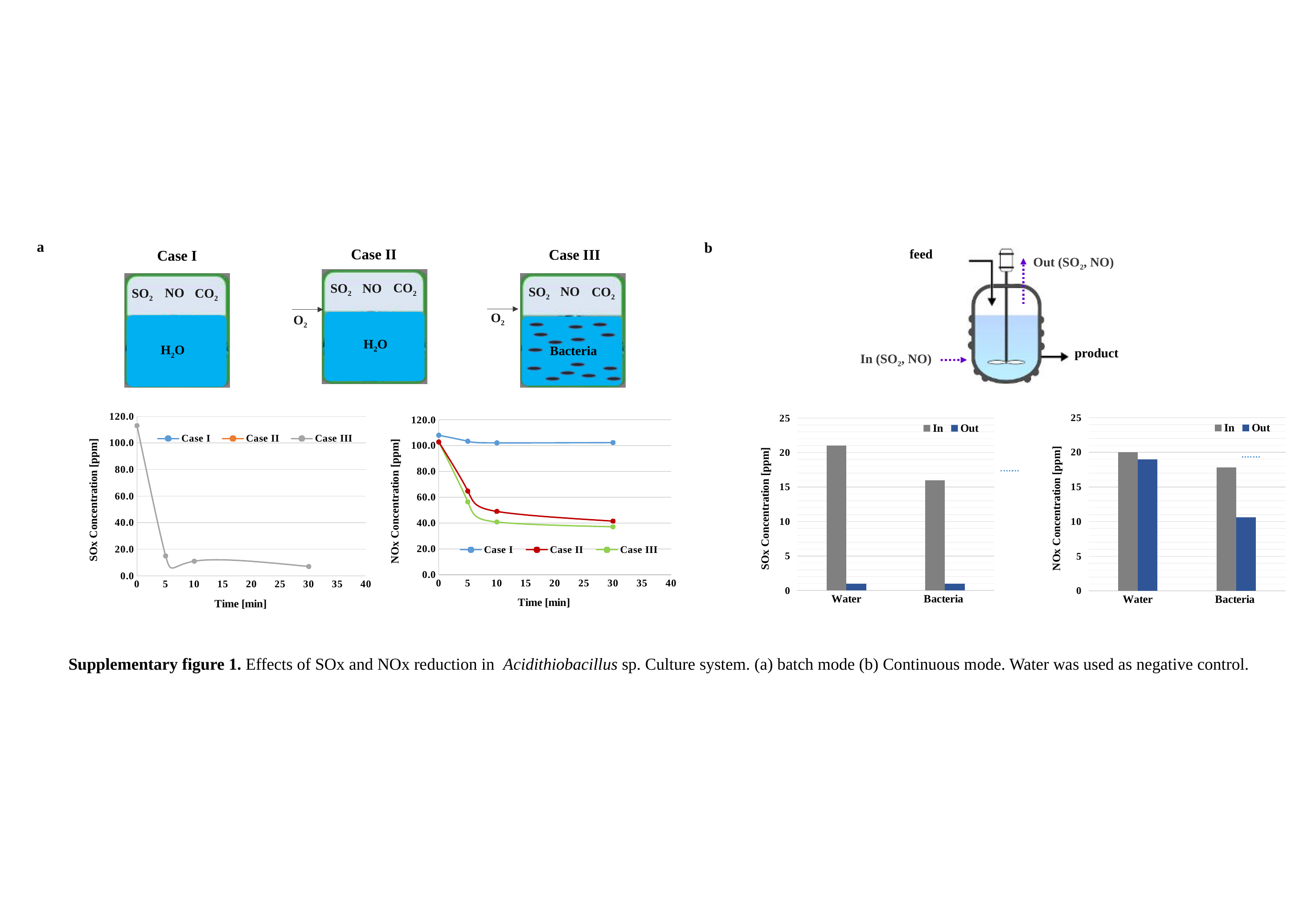

a
b
Case II
Case III
feed
Out (SO2, NO)
product
Case I
CO2
SO2
NO
NO
CO2
SO2
NO
CO2
SO2
O2
O2
H2O
H2O
H2O
Bacteria
In (SO2, NO)
### Chart
| Category | | | |
|---|---|---|---|
### Chart
| Category | | | |
|---|---|---|---|
### Chart
| Category | In | Out |
|---|---|---|
| Water | 20.0 | 19.0 |
| Bacteria | 17.8 | 10.625 |
### Chart
| Category | In | Out |
|---|---|---|
| Water | 21.0 | 1.0 |
| Bacteria | 16.0 | 1.0 |Supplementary figure 1. Effects of SOx and NOx reduction in Acidithiobacillus sp. Culture system. (a) batch mode (b) Continuous mode. Water was used as negative control.
