# Supplementary material for: A bacteria-based carbon sequestration and waste recycling system
Source: Sci Rep. 2022 Jun 28;12:10236. doi: 10.1038/s41598-022-14239-1 (PMC9240062; doi:10.1038/s41598-022-14239-1)
Supplement: Supplementary file 2 — Supplementary Figure 2. [file 41598_2022_14239_MOESM2_ESM.pptx]

## Slide 1
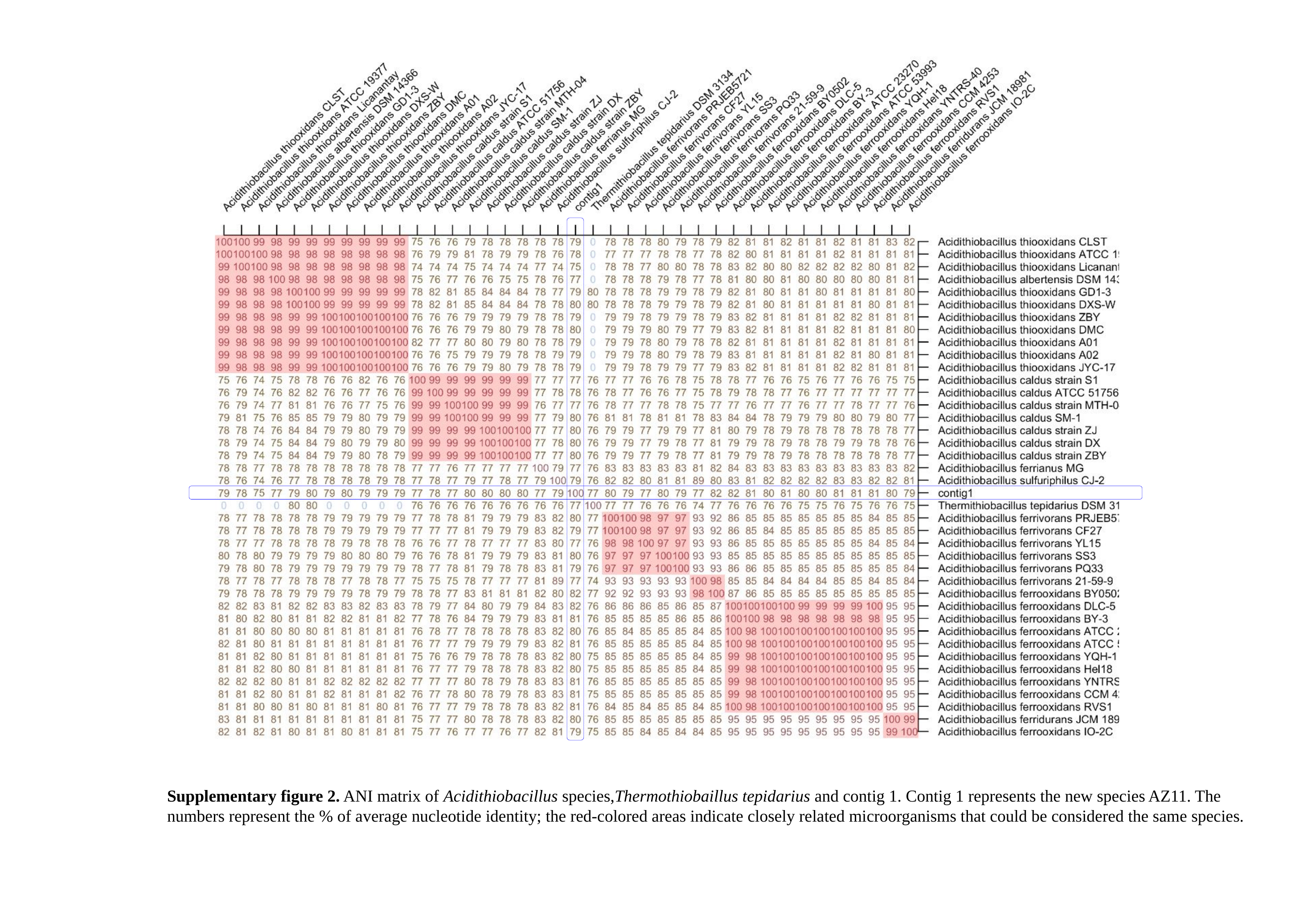

Supplementary figure 2. ANI matrix of Acidithiobacillus species,Thermothiobaillus tepidarius and contig 1. Contig 1 represents the new species AZ11. The numbers represent the % of average nucleotide identity; the red-colored areas indicate closely related microorganisms that could be considered the same species.
